# What Dentists Are Eligible to Membership in the American Medical Association

**Published:** 1894-06

**Authors:** 


### Editorial, Etc.

WHAT DENTISTS ARE ELIGIBLE TO MEMBERSHIP IN THE AMERICAN MEDICAL ASSOCIATION.—The following resolution, introduced by Dr. N. S. Davis, of Chicago, was unanimously passed by the American Medical Association at a recent meeting:

"*Resolved*, That the regular graduates of such dental and oral schools and colleges as require of their students a standard of preliminary or general education and a term of professional study equal to the best class of the medical colleges of this country, and embrace in their curriculum all the fundamental branches of medicine, differing chiefly by substituting practical and clinical instruction in dental and oral medicine and surgery, in place of practical and clinical instruction in general medicine and surgery, be recognized as members of the regular profession of medicine, and eligible to membership in this Association on the same conditions and subject to the same regulations as other members."

